# Chemokine Expression in Inflamed Adipose Tissue Is Mainly Mediated by NF-κB

**DOI:** 10.1371/journal.pone.0066515

**Published:** 2013-06-18

**Authors:** Franck Tourniaire, Beatrice Romier-Crouzet, Jong Han Lee, Julie Marcotorchino, Erwan Gouranton, Jerome Salles, Christiane Malezet, Julien Astier, Patrice Darmon, Eric Blouin, Stephane Walrand, Jianping Ye, Jean-Francois Landrier

**Affiliations:** 1 INRA, UMR 1260, Marseille, France; 2 INSERM, UMR 1062, « Nutrition, Obésité et Risque Thrombotique », Marseille, France; 3 Université d’Aix-Marseille, Faculté de Médecine, Marseille, France; 4 Pennington Biomedical Research Center, Louisiana State University System, Baton Rouge, Louisiana, United States of America; 5 UMR INRA 1019 Unité de nutrition humaine, Centre de Recherches INRA de Clermont-Ferrand/Theix, St Genès Champanelle, France; 6 Laboratoire Labcatal, Montrouge, France; Warren Alpert Medical School of Brown University, United States of America

## Abstract

Immune cell infiltration of expanding adipose tissue during obesity and its role in insulin resistance has been described and involves chemokines. However, studies so far have focused on a single chemokine or its receptor (especially CCL2 and CCL5) whereas redundant functions of chemokines have been described. The objective of this work was to explore the expression of chemokines in inflamed adipose tissue in obesity. Human and mouse adipocytes were analyzed for expression of chemokines in response to inflammatory signal (TNF-α) using microarrays and gene set enrichment analysis. Gene expression was verified by qRT-PCR. Chemokine protein was determined in culture medium with ELISA. Chemokine expression was investigated in human subcutaneous adipose tissue biopsies and mechanism of chemokine expression was investigated using chemical inhibitors and cellular and animal transgenic models. Chemokine encoding genes were the most responsive genes in TNF-α treated human and mouse adipocytes. mRNA and protein of 34 chemokine genes were induced in a dose-dependent manner in the culture system. Furthermore, expression of those chemokines was elevated in human obese adipose tissue. Finally, chemokine expression was reduced by NF-κB inactivation and elevated by NF-κB activation. Our data indicate that besides CCL2 and CCL5, numerous other chemokines such as CCL19 are expressed by adipocytes under obesity-associated chronic inflammation. Their expression is regulated predominantly by NF-κB. Those chemokines could be involved in the initiation of infiltration of leukocytes into obese adipose tissue.

## Introduction

Obesity, which can be defined as an excess of body fat mass, is a major risk for developing type 2 diabetes caused from the systemic insulin resistance. Obesity-induced insulin resistance is believed to result initially from adipose tissue expansion and hypoxia response [1], which leads to the release of free fatty acids (FFAs) into the circulation as well as inducing adipocyte apoptosis or necrosis. On the long term, elevated plasma FFAs contributes to skeletal muscle insulin resistance and augments hepatic glucose production. The importance of adipose tissue has been confirmed by showing that gastric bypass-induced weight loss or surgical removal of excess fat can restore insulin sensitivity in animals and humans [2], [3]. Furthermore, works from Hotamisligil et al. [4] have shown that adipose tissue derived inflammatory mediator Tumor Necrosis Factor-α (TNF-α) is involved in obesity-associated insulin resistance, leading to the “inflammation theory” that suggests that obesity and type 2 diabetes are inflammatory diseases. It has been shown that TNF-α expression is increased in the adipose tissue of obese individuals [4], that its level is correlated with adiposity [5] and numerous studies have highlighted TNF-α involvement in the etiology of insulin resistance [6]. The exact origin of TNF-α remained undetermined until Weisberg et al. and Xu et al. shown that macrophages are infiltrating into adipose tissue in obesity and that macrophages are the major source of TNF-α [7], [8]. These observations greatly enriched the inflammation theory and this finding opened a field of intense research about immune cell infiltration in the adipose tissue.

Macrophage infiltration has been by far the most investigated in obesity, and several groups have studied the role of chemokines (chemoattractant cytokines) such as CCL2/MCP-1 (C-C motif chemokine ligand 2/macrophage chemoattractant protein-1). These studies have shown that inhibition of CCL2 by gene knockout or chemical blockade is able to diminish macrophage infiltration, but unable to block it completely [9], [10], [11], [12], [13], [14], suggesting that other chemokines might be involved in this process. In support of this view, studies have shown that several other chemokines such as CCL5 [15], C-X-C motif chemokine ligand 5 (CXCL5 [16]) and CXCL14 [17] are all involved in adipose macrophage infiltration and pathogenesis of insulin resistance. Again, individual inhibition of the chemokines was not sufficient to completely restore insulin sensitivity.

Actually, almost all types of immune cells (lymphocytes, neutrophils, monocytes/macrophages, dendritic cells, natural killer cells) are infiltrating obese adipose tissue during obesity development [18] and contribute to the pathogenesis of insulin resistance. These studies suggest that insulin resistance generated by obese adipose tissue infiltration relies on several cell types and hence several chemokines. Although the initial event(s) leading to leukocyte infiltration and the exact sequence of infiltration of the different immune cell types remain to be fully established yet, it appears that B cells, T cells and neutrophils would infiltrate at the early stages of adipose tissue expansion, whereas macrophage infiltration would rather happen at the late stages of adipose tissue expansion [19], and contribute to the sustained chronic inflammation [20]. This suggests that adipose infiltration of multiple immune cells is a programmed event. It may be dependent on a network of chemokines, the nature of which remains to be determined.

In this paper, we describe that adipocytes are able to express 34 chemokines involved in the attraction of most immune cells. Furthermore, the role of adipocytes has been confirmed *in vivo*, by measuring chemokine expression in adipose tissue from obese subjects, and we demonstrate that chemokine expression is predominantly regulated via the Nuclear Factor-κB (NF-κB) pathway using chemical inhibitors and transgenic models.

## Materials and Methods

### Reagents

Dulbecco’s modified Eagle’s medium (DMEM) was purchased from Life Technologies (Cergy Pontoise, France), and fetal bovine serum (FBS) was obtained from PAA Laboratories (Les Mureaux, France). Isobutylmethylxanthine, dexamethasone and insulin were purchased from Sigma-Aldrich (Saint Quentin Fallavier, France). TRIzol reagent, random primers and Moloney murine leukemia virus reverse transcriptase were obtained from Life Technologies. SYBR Green reaction buffer was purchased from Eurogentec (Angers, France). Antibodies were purchased from eBiosciences SAS (Paris, France). Unless otherwise specified, all other reagents were purchased from Sigma-Aldrich.

### Cell Culture

3T3-L1 preadipocytes (ATCC, Manassas, VA) were seeded in 3.5-cm diameter dishes at a density of 15×10^4^ cells/well, and grown in DMEM supplemented with 10% FBS, at 37°C in a 5% CO_2_ humidified atmosphere, as previously reported [21]. To induce differentiation, two-day postconfluent 3T3-L1 preadipocytes (day 0) were stimulated for 48 h with 0.5 mM isobutylmethylxanthine, 0.25 µmol/l dexamethasone and 1 µg/ml insulin in DMEM supplemented with 10% FBS. The cultures were then treated with DMEM supplemented with 10% FBS and 1 µg/ml insulin. Then, adipocytes were incubated with TNF-α (15 ng/ml) for 24 h.

Human preadipocytes (isolated from female subcutaneous adipose tissue biopsies) were supplied by Promocell (Heidelberg, Germany), cultured and differentiated into adipocytes according to the company’s instructions. Briefly, cells were seeded at a density of 5000 cells/cm^2^ in Preadipocyte Growth Medium and grown until confluence was reached. Cells were then allowed to differentiate for 3 days in Preadipocyte Differentiation Medium and mature adipocytes were cultivated in Adipocyte Nutrition Medium for 11 additional days. Adipose maturity was assessed at the morphological (light microscopy) and the genomic expression levels (comparison of expression level of pre-adipocyte (Pref-1) and adipocyte markers - CEBPA, aP2, and ADIPOQ -, Figure S1.

In a first series of experiments, mature adipocytes (day 14) were incubated for 24 h with 5–15 ng/ml of TNF-α. In order to identify which pro-inflammatory pathway was involved in TNF-α chemokine expression, mature human adipocytes were pre-incubated for 1 h with either 20 µM JNK-inhibitor-II, 20 µM SB 202190, or 10 µM BAY 11-7082 (c-Jun NH_2_-terminal Kinase – JNK –, p38 mitogen activated protein – MAP – kinase and NF-κB pathway inhibitors, respectively, all obtained from Merck Millipore, Darmstadt, Germany). The medium containing the inhibitor was then aspired; cells were rinsed with PBS and then incubated for 24 h with 15 ng/ml TNF-α in culture medium.

NF-κB p65 null Mouse Embryonic Fibroblasts (MEFs) were obtained from Dr. Inder M. Verma (Salk Institute). The cells were maintained in DMEM cell culture medium supplemented with 10% fetal bovine serum. The cells were treated with TNF-α (20 ng/ml) in serum free medium containing 0.25% BSA for 2 h and total RNA was extracted using TRIzol reagent as described below.

### Hybridization Arrays and Microarray Data Analysis

RNA quality control was performed on an Agilent 2100 Bioanalyzer (Massy, France) with 6000 Nano Chips, according to the manufacturer’s instructions. For human adipocytes and 3T3-L1 cells, six independent cultures were grown in quadruplicate (i.e. 24 controls and 24 TNF-α treated). RNAs were hybridized to Agilent Whole Human Genome (4×44 k; Massy, France). All labeling, hybridization, washing and scanning were performed as described in the manufacturer’s protocol and as previously described [22]. Arrays were scanned with an Agilent Scanner (Massy, France). Data were extracted with Agilent Feature Extraction v10.5.1.1 and analyzed with Agilent GeneSpring GX v11.0.2 (Massy, France). Data were normalized according to the locally weighted scatterplot smoothing (LOWESS) method, and multiple correction test false discovery rate was applied. Further analyses were performed with Gene Set Enrichment Analysis (GSEA) software (http://www.broadinstitute.org/gsea). A False Discovery Rate (FDR) q-value <0.25 for normalized enrichment score was considered significant.

### RNA Isolation and qPCR

Total cellular RNAs were extracted using TRIzol reagent according to the manufacturer’s instructions. cDNAs were synthesized from 1 µg of total RNA using random primers and Moloney murine leukemia virus reverse transcriptase. Real-time quantitative RT-PCR analyses were performed using the Mx3005P Real-Time PCR System (Stratagene, La Jolla, CA) as previously described [23]. For each condition, expression was quantified in duplicate and 18S rRNA was used as the endogenous control in the comparative cycle threshold (C_T_) method. Data were expressed as relative expression ratio. The sequences of the primers and Taqman gene expression assays (Applied Biosystems) used for qPCR determination of gene expression are displayed in Table S1.

### Chemokine Determination in Culture Medium

CCL2 was quantified with the human CCL2 (MCP-1) Ready-SET-Go!® ELISA from eBiosciences. A Milliplex assay (Millipore, Molsheim, France) was used to quantify CCL5, Interleukin-6 (IL-6), CXCL1, CXCL8 and CXCL10 in human adipocyte culture medium with the Luminex 100® platform.

### Human White Adipose Tissue Biopsies

Eleven lean (Body Mass Index: 22.5±0.5 kg/m^2^) and fourteen obese (BMI: 31.7±0.9 kg/m^2^) male subjects were recruited for this study. Lean and obese volunteers were aged 44±7 years and 44±5 years, respectively. Subcutaneous adipose tissue biopsies were performed between 6∶30 a.m. and 7∶30 a.m. after an overnight fast. Biopsies were obtained by needle aspiration in the periumbilical area under local anesthesia. Adipose tissue samples were rinsed in physiologic serum, immediately frozen in liquid nitrogen and stored at −80°C until RNA extraction. The experimental protocol was performed in accordance with the guidelines in the Declaration of Helsinki and was approved by the Ethical Committee of the Auvergne region (agreement no. AU 800; March 2010). Participants provided their written informed consent to participate in this study.

### Animal Experiments

aP2-p65 mice were generated on the C57BL/6J background as described elsewhere [24]. All of the mice were housed in the animal facility at the Pennington Biomedical Research Center with a 12∶12-h light-dark cycle and constant temperature (22–24°C). The male mice were fed chow diet (MF 5001, 11% calorie in fat) and the epididymal fat tissue was collected at 20 weeks. The mice were housed at 4 per cage with free access to water and diet. All procedures were performed in accordance with the National Institutes of Health guidelines for the care and use of animals and were approved by the Institutional Animal Care and Use Committee (IACUC) at the Pennington Biomedical Research Center.

## Results

### TNF-α Induces Chemokine Expression in Adipocytes

Human adipocyte primary cultures were subjected to TNF-α treatment (15 ng/ml) for 24 h after which total RNA was extracted for gene expression analysis using microarrays. Results were validated by qPCR (table S2) of randomly chosen genes, as well as genes whose expression were known to be affected by TNF-α (e.g. *ADIPOQ*, *IRS1* and *PPARG*). Examination of the gene list indicated that several chemokine genes were among the most dramatically upregulated in response to TNF-α: indeed, expression of 34 chemokines were found to be significantly modulated, with *CCL5*, *CCL19* and *CCL20* being the most dramatically affected (Table 1). Gene Set Enrichment Analysis (GSEA) according to gene ontology terms confirmed that chemokines were the most overrepresented genes, and highlighted inflammation related processes such as defense response, locomotory behavior and chemokine activity as the most represented terms (the first 10 most represented gene sets are displayed on Table S3). Furthermore, chemokine expression was found to be dose dependent (Table 1). Interestingly, very similar results were obtained when murine 3T3-L1 differentiated in adipocytes were treated (supplemental tables S4 and S5).

**Table 1:** List of chemokine related genes significantly regulated (p<0.01) by TNF-α treatment in human adipocytes.

			mRNA induction (fold change vs. control)		
Genesymbol	Array Probe	Description	TNF-α 5 ng/ml	TNF-α 10 ng/ml	TNF-α 15 ng/ml
CCL1	A_23_P49759	Homo sapiens chemokine (C-C motif) ligand 1 (CCL1), mRNA [NM_002981]			3.6
CCL13	A_24_P125335	Homo sapiens chemokine (C-C motif) ligand 13 (CCL13), mRNA [NM_005408]			5.1
	A_23_P26965				5.2
CCL17	A_23_P26325	Homo sapiens chemokine (C-C motif) ligand 17 (CCL17), mRNA [NM_002987]	2.6	2.9	4.5
CCL19	A_23_P123853	Homo sapiens chemokine (C-C motif) ligand 19 (CCL19), mRNA [NM_006274]	31.6	46.5	149.9
CCL2	A_23_P89431	Homo sapiens chemokine (C-C motif) ligand 2 (CCL2), mRNA [NM_002982]	10.5	11.5	23.6
CCL20	A_23_P17065	Homo sapiens chemokine (C-C motif) ligand 20 (CCL20), mRNA [NM_004591]	25.6	35.8	145.5
CCL25	A_23_P55828	Homo sapiens chemokine (C-C motif) ligand 25 (CCL25), mRNA [NM_005624]			1.4
CCL28	A_23_P503072	Homo sapiens chemokine (C-C motif) ligand 28 (CCL28), mRNA [NM_148672]			1.8
CCL5	A_23_P152838	Homo sapiens chemokine (C-C motif) ligand 5 (CCL5), mRNA [NM_002985]	49.0	64.1	344.8
CCL7	A_23_P78037	Homo sapiens chemokine (C-C motif) ligand 7 (CCL7), mRNA [NM_006273]	3.9	4.5	4.8
CCL8	A_23_P207456	Homo sapiens chemokine (C-C motif) ligand 8 (CCL8), mRNA [NM_005623]	18.8	26.7	26.0
CMKLR1	A_23_P105465	Homo sapiens chemokine-like receptor 1 (CMKLR1), mRNA [NM_004072]	−3.6	−4.1	−3.7
	A_23_P105461		−10.2	−8.9	−12.8
	A_24_P766716		−14.1	−18.7	−21.8
CCR1	A_24_P148717	Homo sapiens chemokine (C-C motif) receptor 1 (CCR1), mRNA [NM_001295]	−3.0	−2.0	−2.7
CCR7	A_23_P343398	Homo sapiens chemokine (C-C motif) receptor 7 (CCR7), mRNA [NM_001838]			1.3
CCRL1	A_23_P6909	Homo sapiens chemokine (C-C motif) receptor-like 1 (CCRL1), transcript variant 1, mRNA [NM_178445]	−3.1	−3.7	−4.4
CCRL2	A_23_P69310	Homo sapiens chemokine (C-C motif) receptor-like 2 (CCRL2), mRNA [NM_003965]			2.1
CX3CL1	A_24_P381901	Homo sapiens chemokine (C-X3-C motif) ligand 1 (CX3CL1), mRNA [NM_002996]			3.6
	A_24_P390495		5.5	10.0	34.0
	A_23_P37727		19.1	34.4	87.8
CXCL1	A_23_P7144	Homo sapiens chemokine (C-X-C motif) ligand 1 (melanoma growth stimulating activity, alpha) (CXCL1), mRNA [NM_001511]	14.8	17.1	35.3
CXCL10	A_24_P303091	Homo sapiens chemokine (C-X-C motif) ligand 10 (CXCL10), mRNA [NM_001565]	12.4	17.6	34.0
CXCL11	A_24_P20607	Homo sapiens chemokine (C-X-C motif) ligand 11 (CXCL11), mRNA [NM_005409]	6.3	7.7	32.2
	A_23_P125278		21.1	37.8	113.9
CXCL12	A_23_P202448	Homo sapiens chemokine (C-X-C motif) ligand 12 (stromal cell-derived factor 1) (CXCL12), transcript variant 1, mRNA [NM_199168]	2.3	2.0	
	A_24_P944054		4.5	3.3	1.8
	A_24_P412156		5.2	4.0	3.4
CXCL13	A_23_P121695	Homo sapiens chemokine (C-X-C motif) ligand 13 (B-cell chemoattractant) (CXCL13),mRNA [NM_006419]			10.8
CXCL14	A_23_P213745	Homo sapiens chemokine (C-X-C motif) ligand 14 (CXCL14), mRNA [NM_004887]			3.4
CXCL16	A_23_P38505	Homo sapiens chemokine (C-X-C motif) ligand 16 (CXCL16), mRNA [NM_022059]			1.4
CXCL2	A_23_P315364	Homo sapiens chemokine (C-X-C motif) ligand 2 (CXCL2), mRNA [NM_002089]	14.1	15.1	15.3
	A_24_P257416		5.7	6.4	12.1
CXCL3	A_24_P183150	Homo sapiens chemokine (C-X-C motif) ligand 3 (CXCL3), mRNA [NM_002090]	15.1	16.7	32.4
	A_24_P251764		3.2	3.2	5.6
CXCL5	A_23_P110204	Homo sapiens chemokine (C-X-C motif) ligand 5 (CXCL5), mRNA [NM_002994]	22.9	27.7	24.0
	A_24_P277367		9.5	10.7	11.0
CXCL6	A_23_P155755	Homo sapiens chemokine (C-X-C motif) ligand 6 (granulocyte chemotactic protein 2)(CXCL6), mRNA [NM_002993]	32.1	38.2	53.8
CXCL8	A_32_P87013	Homo sapiens interleukin 8 (IL8), mRNA [NM_000584]	22.8	24.6	70.8
CXCL9	A_23_P18452	Homo sapiens chemokine (C-X-C motif) ligand 9 (CXCL9),mRNA [NM_002416]			5.1
CXCR2	A_23_P135755	Homo sapiens chemokine (C-X-C motif) receptor 2 (CXCR2), transcript variant 1, mRNA [NM_001557]			−1.9
DARC	A_23_P115161	Homo sapiens Duffy blood group, chemokine receptor (DARC), mRNA [NM_002036]			1.4
FAM19A2	A_24_P297551	Homo sapiens family with sequence similarity 19 (chemokine (C-C motif)-like), member A2 (FAM19A2), mRNA [NM_178539]			−2.7
FAM19A5	A_23_P304489	Homo sapiens family with sequence similarity 19 (chemokine (C-C motif)-like), member A5 (FAM19A5), transcript variant 2, mRNA [NM_015381]	−2.1	−2.2	−1.8

**Figure 1:**
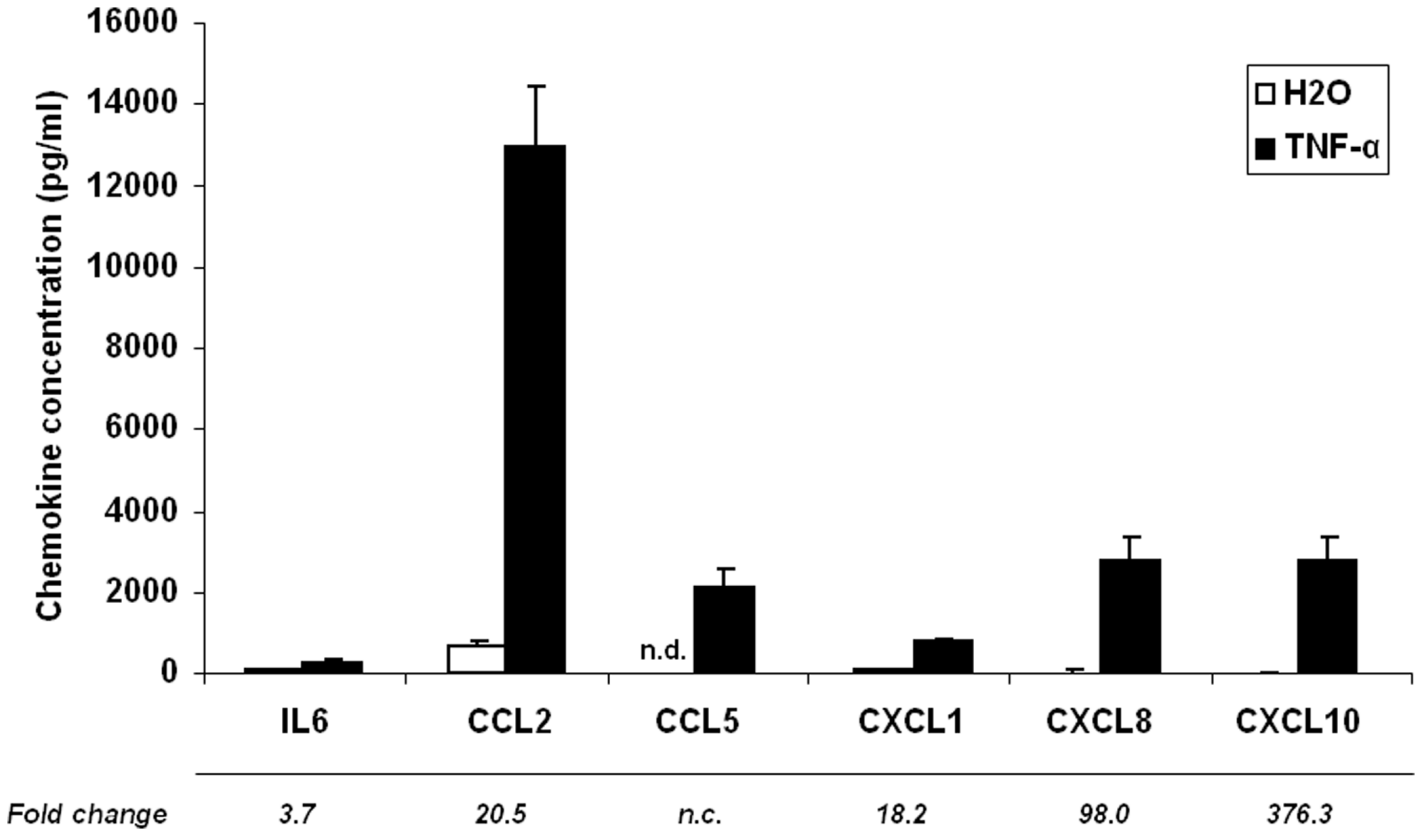
TNF-α induces chemokine secretion in the culture medium of human adipocytes. Cells were incubated for 24 h with TNF-α (15 ng/ml). White diagrams represent the control value and black diagram the TNF-α treatment value. Data are shown as mean ± SEM of 6 independent experiments. *n.d.: not detected.*

A parallel increase in protein secretion in culture medium was investigated for a subset of these chemokines (Figure 1). Again, CCL5 was found to be the most significantly induced protein, being undetectable in non stimulated adipocytes, and reaching a concentration of around 2000 pg/ml after 24 h of TNF-α treatment. CXCL8 and CXCL10 levels were also dramatically increased (by 98.0 and 376.3 fold, respectively) in response to TNF-α. CCL2, CXCL1 and IL-6 secretion were also increased in the culture medium, but to a lesser extent.

### Chemokine Expression is Upregulated in the Adipose Tissue of Obese Individuals

**Figure 2:**
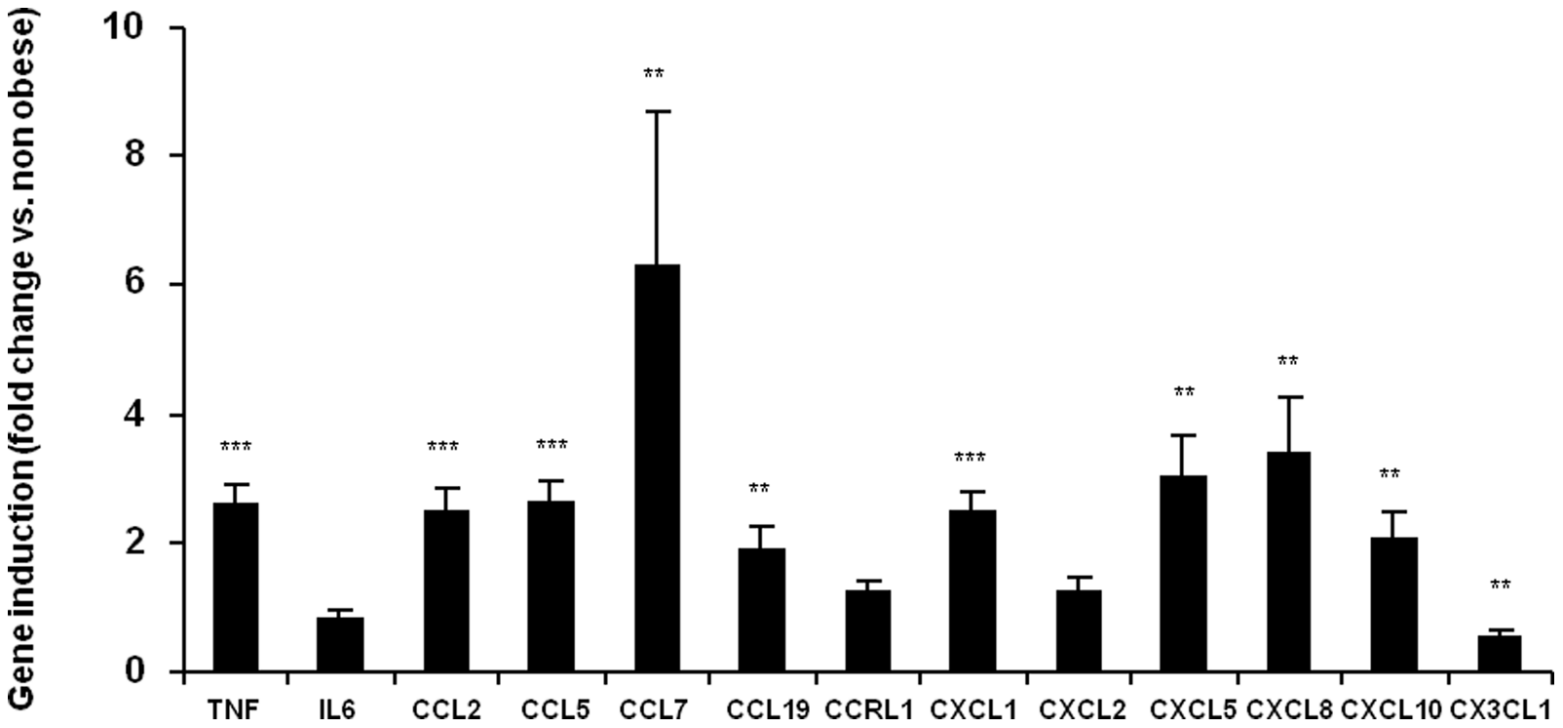
Chemokine expression is increased in obese adipose tissue from obese subjects. Chemokine mRNA expression levels were analyzed by qPCR. Data are shown as mean ± SEM, (N = 11–14 volunteers/group). *t-test significance:* *p<0.05, **p<0.01, ***p<0.001.

To confirm our observations, chemokine expression was investigated in subcutaneous adipose tissue of obese and lean subjects (Figure 2). A significant increase in expression was observed in most of the genes investigated: *CCL2*, *CCL5*, *CCL7*, *CCL19*, *CXCL1*, *CXCL5*, *CXCL8* and *CXCL10* were increased by 2.5, 2.7, 1.9, 6.3, 2.5, 3.0, 3.4 and 2.0 fold, respectively, in obese subjects vs. controls. *CCRL1* (C-C motif chemokine receptor-like 1) and *CXCL2*, albeit being overexpressed in response to TNF-α *in vitro* had their level unchanged in obese adipose tissue. Surprisingly, *CX3CL1* (C-X3-C motif ligand 1) expression was found to be 1.7 fold lower in obese subjects (Figure 2).

Additionally, we observed that *TNF* expression was higher in the adipose tissue of obese subjects, and was significantly correlated with the expression of *CCL2*, *CCL5*, *CCL7*, *CXCL1*, *CXCL2* and *CXCL8* (Table 2).

**Table 2:** Correlation between TNF and chemokine expression levels in human adipose tissue.

Gene	CCL2	CCL5	CCL7	CCL19	CCRL1	CXCL1	CXCL2	CXCL5	CXCL8	CXCL10	CX3CL1
R^a^	0.740	0.686	0.612	0.168	−0.037	0.782	0.454	0.117	0.338	0.460	−0.230
P value	*<0.001*	*<0.001*	*<0.001*	*0.244*	*0.797*	*<0.001*	*0.001*	*0.427*	*0.016*	*0.001*	*0.108*

a Pearson correlation coefficient, N = 25.

### TNF-α Induced Chemokine Expression is Mediated by NF-κB

To unravel the molecular mechanisms underlying chemokine expression in adipocytes, we analyzed the microarray data with GSEA again, but this time according to transcription factor binding site overrepresentation (Table S6). Interestingly, we found that NF-κB binding site as the most enriched response element, which is fully consistent with the fact NF-κB plays a central role in the regulation of inflammatory response and is known to be activated by TNF-α [25]. Similar data were obtained for TNF-α -treated 3T3-L1 cells (Table S7). Furthermore, similarly to what Ruan et al. [26] reported in 3T3-L1 cells, we observed an increase in mRNA levels of several members of the NF-κB family such as *NFKB1* (×3.2), *NFKB2* (×5.8), *RELA* (×1.6), *RELB* (×9.9) and associated regulatory proteins such as inhibitor of kappa light polypeptide gene enhancer in B-cells, kinase beta, *IKBKB* (×1.3), *IKBKE* (×4.4), *IKBKG* (×1.1) and B-cell CLL/lymphoma 3, *BCL3* (×2.5) following TNF-α treatment.

These data reinforce the putative major role of NF-κB in the regulation of chemokine expression under these conditions.

**Figure 3:**
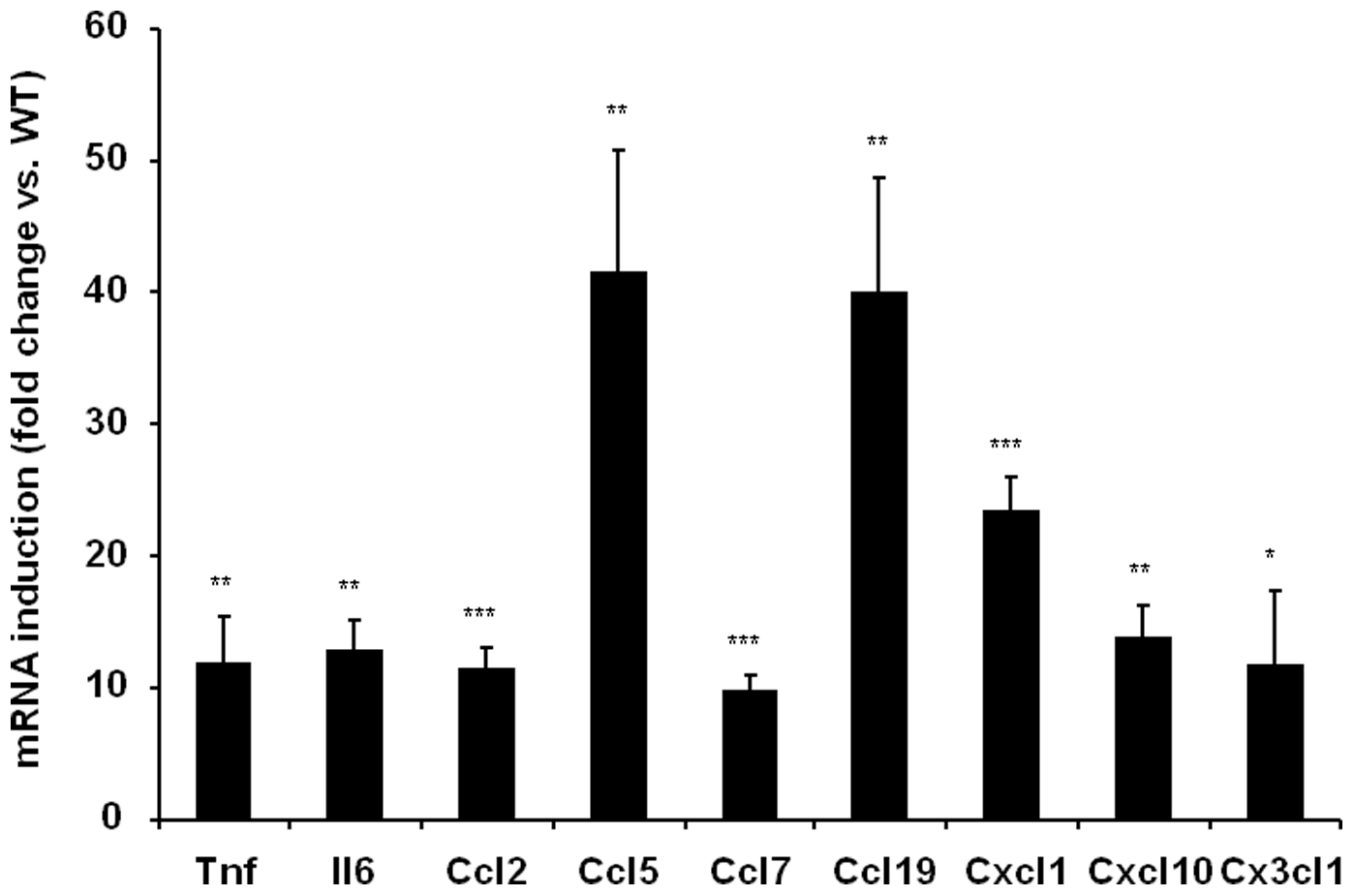
Chemokine expression is induced in the adipose tissue of aP2-p65 mice. Chemokine expression levels were compared with that of WT animals (mean ± SEM, N = 3 animals/group). *t-test significance:* *p<0.05, **p<0.01, ***p<0.001.

To confirm the involvement of NF-κB in the regulation of chemokine expression *in vivo*, we took advantage of a murine model in which the p65 gene, encoding the transcriptional activity of NF-κB, has been overexpressed (Figure 3). The expression of all chemokines assessed by qPCR was found to be increased in the white adipose tissue of aP2-p65 mice by at least 9.5-fold, with *Ccl5* and *Ccl19* being increased by around 40-fold. *Ccl20* mRNA was detected in the adipose tissue of aP2-p65 mice only, with a mean C_T_ value of 29.5.

**Figure 4:**
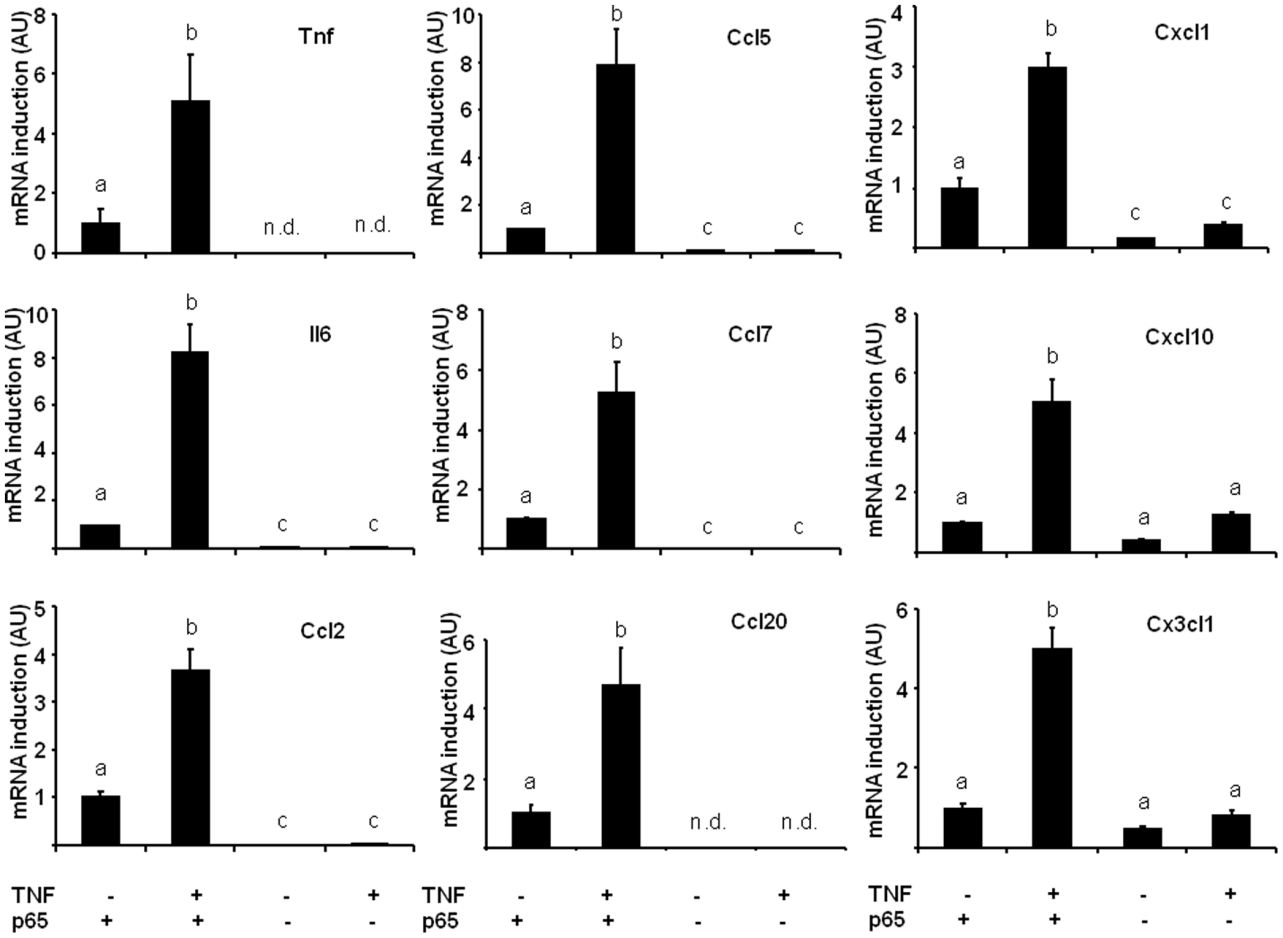
Chemokine expression in response to TNF-α is highly dependent on p65 NF-κB activity. Gene expression was measured by qPCR in WT or p65 null MEFs treated or not with TNF-α (mean ± SEM of 4–5 independent experiments). *n.d.: not detected, values sharing a different letter are statistically different at p<0.05 (one-way ANOVA followed by Tukey-Kramer post hoc test).*

The implication of NF-κB was then assessed in *p65* null MEFs: basal chemokine expression in untreated cells was always lower in *p65* null MEFs than in wild-type cells, suggesting that NF-κB is involved in the constitutive expression of these chemokines (Figure 4). On the other hand, TNF-α treatment of WT MEF cells resulted in the upregulation of *Tnf*, *Il6* and chemokine mRNA expression by at least 3.5 fold (except *Ccl19* which could not be detected, Figure 4). As expected, no statistically significant induction could be observed in p65 null cells in response to TNF-α, and *Tnf* and *Ccl20* expression became undetectable.

These data indicate that NF-κB is the main mediator of chemokine expression in response to TNF-α. Still, a noticeable but non significant induction was observed for *Cxcl1*, *Cxcl10* and *Cx3cl1* in response to TNF-α in *p65* null MEF cells. This observation suggests that there might be an alternative signaling pathway mediating TNF-α induced chemokine expression.

**Figure 5:**
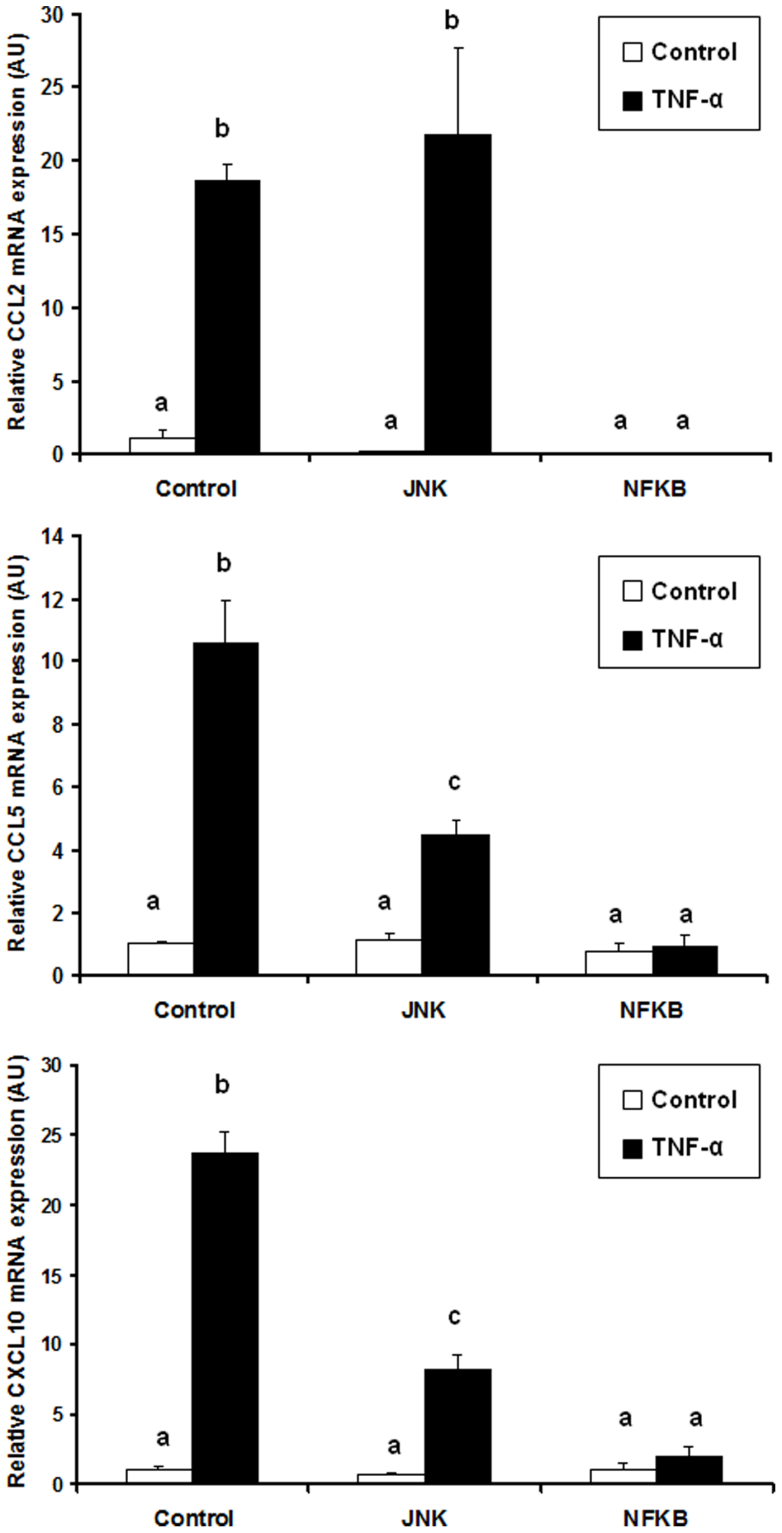
Effect of JNK and NF-κB inhibitors on TNF-α induced chemokine expression in human adipocytes. Chemokine mRNA expression levels were analyzed by qPCR. Data are shown as mean ± SEM, (N = 3). *Values sharing a different letter are statistically different at p<0.05 (one-way ANOVA followed by Tukey-Kramer post hoc test).*

Finally, to investigate the possible involvement of other signaling pathways in TNF-α induced chemokine expression in adipocytes, we preincubated cells with chemical inhibitors of p38, JNK or NF-κB before performing TNF-α stimulation (15 ng/ml). The expression level of *CCL2*, *CCL5* and *CXCL10* was then measured using qPCR. While p38 chemical blockade did not modify chemokine expression in response to TNF-α (data not shown), JNK inhibition led to a decrease in CCL5 and CXCL10 expression, whereas CCL2 level was not affected (Figure 5). Finally, inhibition of the NF-κB pathway totally suppressed chemokine expression in response to TNF-α.

Altogether, these results clearly indicate that NF-κB is the central regulator of chemokine expression in response to TNF-α.

## Discussion

Obesity is associated with an increased risk of type 2 diabetes or cardiovascular diseases. Studies over the past 10 years have highlighted the role of inflammatory mediators secreted by the adipose tissue (such as chemokines and cytokines) in the incidence of systemic insulin resistance [27]. Although adipose tissue inflammation is related to infiltration of multiple immune cells, the underlying mechanism is largely unknown. In this study, we addressed this question by investigating chemokine network in adipocytes and adipose tissue.

Chemokines are defined as “cytokines with selective chemoattractant properties”, coordinating the leukocyte movement to sites of inflammation or injury [28]. The chemokine system, which comprises around 50 ligands and 20 receptors, is characterized by its redundancy [29], i.e. some chemokines share a common receptor, and most receptors are able to interact with several ligands. So far, only a few chemokines have been investigated for their role in obesity for leukocyte infiltration, and several chemokines have been reported in the etiology of obesity-associated inflammation and insulin resistance [9], [11], [15], [16], [17]. Whereas most of the attention is focused on CCL2/MCP-1 (refs 10, 32), it is however noteworthy that knockout or blocking of CCL2 or CCR2 failed (when having any effect) to completely suppress macrophage infiltration into adipose tissue or fully to restore insulin sensitivity [9], [13], [14], [30]. Similar modest effects have also been observed in intervention of chemokines such as CCL3, CXCL5, or CXCL14 (refs 16, 17, 34). This group of studies strongly suggests that other chemokines might compensate the loss of one chemokine in these models and that the chemokine network have to be taken into account when studying immune cell infiltration in the adipose tissue. In agreement with this assumption, the ability of subcutaneous adipose tissue to express multiple chemokine ligands and receptors has been reported in humans [31], [32], [33], [34]. In addition, in the present study we showed *in vitro* that human adipocytes are able to express and probably secrete a large range of chemokines (Table 1, Figure 1). Furthermore, the fact that very similar observations were obtained with the 3T3-L1 cell line rules out the possibility of a significant contribution from other cell types to chemokine expression in human primary cultures. Altogether, these data support that adipocytes produce a range of chemokines and are important to determine the function of chemokine network in the adipose tissue. Finally, we demonstrated that expression of these chemokines is elevated in obese condition in subcutaneous adipose tissue (Figure 2), which is in agreement with previous observations from Dahlman et al. [10] and Huber et al [32]. However, it should be noted that the most upregulated genes in response to TNF-α in vitro (i.e. CCL5, CCL19 and CCL20) are not the ones that are overexpressed in obese vs. lean subjects. Discrepancies between the results observed in cell culture and adipose tissue biopsies could arise from 1) methodological aspects: in cell culture only adipocytes were present whereas biopsies also contain other cell types in which gene regulation might be different to what is occurring in adipocytes and therefore could dilute or mask gene regulation in the adipocyte fraction. Furthermore, gene expression results were generated using microarrays in the case of adipocytes whereas qPCR was used to compare chemokine expression between obese and lean adipose tissue, which makes the comparison of data in terms of fold change impossible. 2) biological aspects: the in vitro experiment is an acute inflammatory signal (24 h) whereas in the obese biopsies low grade inflammation might have been present for years. Hence, the in vitro experiment might reflect the early phases of chemokine expression by adipocytes whereas in vivo data should be regarded as the chemokine expression pattern resulting from established obesity (probably several years).

If the exact origin of leukocyte infiltration remains elusive (although it has been proposed to result from ceramide production due to excess lipid intake leading to subsequent TNF-α synthesis [35], the chronology of adipose tissue infiltration by leukocytes would first see the arrival of neutrophils, B cells and T cells before monocytes/macrophages [36]. Of interest regarding the initiation of adipose tissue infiltration, CXCL1, CXCL3, CXCL5, CXCL6, and CXCL8 are known to be able to mediate the recruitment of neutrophils (through binding to CXCR1 and/or 2 [28]), which would be the earliest type of leukocytes detected in adipose tissue in response to high fat feeding in mice [37]. Furthermore, the involvement of CXCL5 in adipose tissue macrophage infiltration and muscle insulin resistance has already been described [16]. Microarray data also revealed that *CCL19* and *CCL20* were among the most upregulated genes. Interestingly, these are ligands of the CCR7 and CCR6 receptors [28], respectively, and are expressed by B lymphocytes. Since B cells are also detected early in the adipose tissue of HFD fed animals [36] and have been proposed as important players in insulin resistance development [38], this strengthens the idea that adipocytes could be primers of the adipose tissue inflammation. Furthermore, CCL20 is able to attract Th17 cells, whose implication is increasingly studied in pathological inflammation states through their ability to secrete the pro-inflammatory cytokine IL17 [39]. Duffaut et al. reported that *CCL20* expression is associated with BMI, is elevated in adipocytes from obese subjects and mediates lymphocyte migration [31]. We also found that CCL19 expression is increased in human adipose. To our knowledge, this is the first time that this gene is reported for being overexpressed in obese adipose tissue.

The role of infiltrated T cells in the generation of insulin resistance is also increasingly studied [40]. Interestingly, among the most overexpressed chemokines highlighted in our study, several of them are able to promote the attraction of Th1 (pro-inflammatory) cells (i.e. CXCL10-11 via CXCR3 binding, CCL5 and CCL8 via CCR5, CCL19 via CCR7 and CX3CL1 via binding to CX3CR1). Similar data were observed in vivo for CCL5, CCL19 and CXCL10 in obese adipose tissue (Figure 2).

Finally, human adipocytes were found to strongly express chemokines able to stimulate monocyte attraction in response to TNF-α, especially CXCL1-3, 5–6, 8, and 10–11, which are ligands for CXCR1 and/or 2, but also CCL2 via CCR2, CCL5 via CCR1 or CCR5, CCL8 via CCR or CCR5 binding and CX3CL1 via CX3CR1 binding [28]. In line with previous reports [15], [32], CCL2 and CCL5 were also more expressed in obese compared to lean adipose tissue (Figure 2). Elevated circulating levels of CX3CL1 (fractalkine) have been recently reported in type 2 diabetes subjects [41]. Even though CX3CL1 expression was found to be regulated by TNF-α in human adipocytes, its expression decreased in the adipose tissue of obese vs. lean volunteers (Figure 2). Whereas Shah et al. showed that CX3CL1 was more expressed in visceral vs. subcutaneous fat [41], to our knowledge no data are available regarding its expression in human subcutaneous obese vs. lean subjects. It is therefore possible that CX3CL1, even if regulated by TNF-α in vitro, is not differently expressed in the subcutaneous adipose tissue of obese and lean individuals. The origin of this discrepancy will require further experiments.

TNF-α secretion is central in maintaining obesity driven inflammatory loop as it is produced by infiltrated leukocytes from the stromal fraction [42], and we found that its expression level in adipose tissue was correlated with those of many chemokines in adipose tissue biopsies (Table 2). In our in vitro experiments, TNF-α was used to mimic mild inflammatory conditions, as described previously [43]. To find out how TNF-α regulates chemokine expression we ran GSEA analysis of the microarray data, which indicated NF-κB as the main response element present in the overrepresented genes (Table S6). Then, to confirm experimentally the involvement of NF-κB in the regulation of chemokines, we used 2 transgenic models: adipose tissue from aP2-p65 overexpressing mice (Figure 3), and MEFs invalidated for p65 gene (Figure 4), the latter being treated or not with TNF-α. All chemokine genes were overexpressed in aP2- p65 adipose tissue and lost their TNF-α dependent expression in p65-null cells. However, the existence of a residual albeit non significant gene induction following TNF-α treatment was observed for *Cxcl1*, *Cxcl10* and *Cx3cl1* in p65 null MEFs. This effect could be explained by the fact that TNF-α is also able to mediate its pro-inflammatory effects, besides NF-κB, via another signaling pathways such as p38 or JNK. Indeed, pretreatment of adipocytes with a JNK inhibitor before TNF-α treatment reduced CCL5 and CXCL10 expression in human adipocytes whereas inhibition of NF-κB completely suppressed TNF-α chemokine upregulation (Figure 5). Our results are in agreement with studies from Jiao and colleagues, who identified six CCLs (i.e. CCL2, CCL3, CCL6, CCL7, CCL8 and CCL9) upregulated in the adipose tissue of HFD induced and genetically obese mice [44]. They observed that FFAs could induce the expression of these chemokines in 3T3-L1 adipocytes and that JNK inhibition could only limit the expression of MCP-1/CCL2 and MCP-3/CCL7 whereas NF-κB inhibition was able to repress the expression of all the chemokines tested. These data are similar to what we observed in TNF-α treated adipocytes and support the fact that NF-κB is the major regulator of chemokine expression in response to pro-inflammatory molecules even if redundant signalling pathways could also participate.

In conclusion, our results show that adipocytes are intrinsically able to initiate adipose tissue infiltration by all subtypes of leukocytes through the secretion of multiple chemokines under the effect of TNF-α, via a NF-κB dependent pathway. Also, the high number of chemokines induced (most of which have redundant properties) supports the fact that leukocyte adipose tissue infiltration observed during obesity is not related to one or two proteins such as CCL2 or CCL5. We propose that less ‘classical’ chemokines should be investigated for their role in the development of insulin resistance through recruitment of leukocytes in the adipose tissue and their use as alternative or more sensitive markers of infiltration. In particular, it would be of interest to investigate in more details the role of CCL19, as the expression level of this chemokine in adipocytes was found to be highly sensitive to TNF-α as well as adiposity.

## Supporting Information

Figure S1 **Monitoring of human pre-adipocyte differentiation.**
(DOC)

Table S1 **Sequence of the primers and references of the TaqMan® Gene Expression Assays used for qPCR.**
(DOC)

Table S2 **qPCR validation of microarray data.**
(DOC)

Table S3 **Gene set enrichment analysis of TNF-α treated human adipocyte microarray data according to gene ontology.**
(DOC)

Table S4 **List of chemokine related genes significantly regulated by TNF-α treatment in 3T3-L1 adipocytes.**
(DOC)

Table S5 **Gene set enrichment analysis of TNF-α treated 3T3-L1 microarray data according to gene ontology.**
(DOC)

Table S6 **Gene set enrichment analysis of TNF-α treated human adipocyte microarray data according to transcription factor response element present in the gene promoter.**
(DOC)
