# Supplementary material for: Chemokine Expression in Inflamed Adipose Tissue Is Mainly Mediated by NF-κB
Source: PLoS One. 2013 Jun 18;8(6):e66515. doi: 10.1371/journal.pone.0066515 (PMC3688928; doi:10.1371/journal.pone.0066515)
Supplement: Figure S1 — Monitoring of human pre-adipocyte differentiation. (DOC) [file pone.0066515.s001.doc]

**A**

**B**

**C**

*

*

*

*

**Figure S1. Monitoring of human pre-adipocyte differentiation.** Acquisition of adipocyte characteristics was checked by optical observation (10 times magnification) of cell culture (panels A: pre-adipocyte stage; panel B: differentiated adipocytes) as well as qPCR measurement of differentiation markers (panel C). Data are shown as mean ± SEM, (N=3/group).

** t-test significant at p<0.006*
