# Supplementary material for: Chemokine Expression in Inflamed Adipose Tissue Is Mainly Mediated by NF-κB
Source: PLoS One. 2013 Jun 18;8(6):e66515. doi: 10.1371/journal.pone.0066515 (PMC3688928; doi:10.1371/journal.pone.0066515)
Supplement: Table S1 — Sequence of the primers and references of the TaqMan® Gene Expression Assays used for qPCR. (DOC) [file pone.0066515.s002.doc]

Table S1. Sequence of the primers and references of the TaqMan® Gene Expression Assays used for qPCR.

| Gene Symbol | Primer name | 5’→3’ primer sequence |
| --- | --- | --- |
| Il6 | mIl6_F | acaagtcggaggcttaattacacat |
|  | mIl6_R | ttgccattgcacaactcttttc |
| Cxcl10 | mCxcl10_F | gctgccgtcattttctgc |
|  | mCxcl10_R | tctcactggcccgtcatc |
| Cx3cl1 | mCx3cl1_F | catccgctatcagctaaacca |
|  | mCx3cl1_R | cagaagcgtctgtgctgtgt |
| Ccl2 | mCcl2_F | catccacgtgttggctca |
|  | mCcl2_R | gatcatcttgctggtgaatgagt |
| Ccl5 | mCcl5_F | tgcagaggactctgagacagc |
|  | mCcl5_R | gagtggtgtccgagccata |
| Ccl7 | mCcl7_F | ccaccatgaggatctctgc |
|  | mCcl7_R | ttgacatagcagcatgtggat |
| Tnf | mTnf_F | catcttctcaaaattcgagtgacaa |
|  | mTnf_R | tgggagtagacaaggtacaaccc |
| Rps18 | Rps18_F | cgccgctagaggtgaaattct |
|  | Rps18_R | cattcttggcaaatgctttcg |
| CX3CL1 | hCX3CL1_F | ccaccttctgccatctgac |
|  | hCX3CL1_R | atgttgcatttcgtcacacc |
| CXCL2 | hCXCL2_F | cgcccatggttaagaaaatc |
|  | hCXCL2_R | aggaacagccaccaataagc |
| CXCL5 | hCXCL5_F | ccttttctaaagaaagtcatccaga |
|  | hCXCL5_R | tgggttcagagacctccaga |
| CXCL8 | hCXCL8_F | agacagcagagcacacaagc |
|  | hCXCL8_R | cacagtgagatggttccttcc |
| CCRL1 | hCCRL1_F | agccaaccagtacttttagcattta |
|  | hCCRL1_R | gaaagcatcattcatatgtatccaa |
| CCL19 | hCCL19_F | tcagcctgctggttctctg |
|  | hCCL19_R | gcagtcttcagcatcattgg |
| IRS1 | hIRS1_F | tatgccagcatcagtttcca |
|  | hIRS1_R | tttgctgaggtcatttaggtctt |
| PLIN1 | hPLIN1_F | acattaaagggaagaagttgaagc |
|  | hPLIN1_R | ttctcctgctcagggaggt |
| RXRA | hRXRA_F | acatgcagatggacaagacg |
|  | hRXRA_R | gagagccccttggagtcag |
| SLC2A4 | hSLC2A4_F | ctgtgccatcctgatgactg |
|  | hSLC2A4_R | cgtagctcatggctggaact |
| TNF | hTNF_F | gccagagggctgattagaga |
|  | hTNF_R | cagcctcttctccttcctgac |
| CXCL10 | hCXCL10_F | gaaagcagttagcaaggaaaggt |
|  | hCXCL10_R | gacatatactccatgtagggaagtga |
| CXCL1 | hCXCL1_F | catcgaaaagatgctgaacagt |
|  | hCXCL1_R | ataagggcagggcctcct |
| IL6 | hIL6_F | gcccagctatgaactccttct |
|  | hIL6_R | gaaggcagcaggcaacac |
| CCL2 | hCCL2_F | ttctgtgcctgctgctcat |
|  | hCCL2_R | ggggcattgattgcatct |
| CCL5 | hCCL5_F | cctcattgctactgccctct |
|  | hCCL5_R | ggtgtggtggtccgaggaata |
| CCL19 | hCCL19_F | tcagcctgctggttctctg |
|  | hCCL19_R | gcagtcttcagcatcattgg |
| CEBPA | hCEBPA_F | ccctcagccttgtttgtactgtatg |
|  | hCEBPA_R | ttcgtgttcctaggcaatgct |
| PREF-1 | hPREF1_F | gaacatctctatcacagagctccc |
|  | hPREF1_R | aacccgggcagtgca |
| aP2 | haP2_F | atatgaaagaagtaggagtgggcttt |
|  | haP2_R | ccatgccagccactttcc |
| ADIPOQ | hADIPOQ_F | cctggtgagaagggtgagaa |
|  | hADIPOQ_R | caccgatgtctcccttagga |
|  | | Taqman assay reference |
| Ccl19 |  | Mm00839967_g1 |
| Ccl20 |  | Mm01268754_m1 |
| Cxcl1 |  | Mm00433859_m1 |
| Rps18 |  | Mm00507222_s1 |
| CCL7 |  | Hs00171147_m1 |
| CCL20 |  | Hs01011368_m1 |
