# Supplementary material for: Chemokine Expression in Inflamed Adipose Tissue Is Mainly Mediated by NF-κB
Source: PLoS One. 2013 Jun 18;8(6):e66515. doi: 10.1371/journal.pone.0066515 (PMC3688928; doi:10.1371/journal.pone.0066515)
Supplement: Table S2 — qPCR validation of microarray data. (DOC) [file pone.0066515.s003.doc]

Table S2. qPCR validation of microarray data.

| **Gene name** | **Fold change qPCR** | **p-value fold change qPCR (t-test)** | **Fold change microarray** |
| --- | --- | --- | --- |
| CCL5 | 22.7 | 7.311×10^-7^ | 344.8 |
| CCL19 | 3466 | 1.635×10^-7^ | 149.9 |
| CCRL1 | -2.9 | 0.001 | -4.4 |
| CX3CL1 | 6.2 | 2.806×10^-9^ | 3.6 - 87.8 |
| CXCL2 | 16.9 | 9.371×10^-6^ | 13.7 |
| CXCL5 | 26.9 | 2.037×10^-6^ | 17.5 |
| IL6 | 8.2 | 7.814×10^-8^ | 6.8 |
| CCL2 | 30.0 | 2.250×10^-7^ | 23.6 |
| ADIPOQ | -4.2 | 0.004 | -4.9 |
| IRS1 | -4.3 | 4,093×10^-8^ | -3.5 |
| PLIN1 | -5.3 | 2.820×10^-5^ | -4.7 |
| PPARG | -2.0 | 0.003 | -2.3 |
| RXRA | -1.4 | 0.0769 | -1.6 |
| SLC2A4 | -6.7 | 1.425×10^-5^ | -7.35 |
