# Supplementary material for: Chemokine Expression in Inflamed Adipose Tissue Is Mainly Mediated by NF-κB
Source: PLoS One. 2013 Jun 18;8(6):e66515. doi: 10.1371/journal.pone.0066515 (PMC3688928; doi:10.1371/journal.pone.0066515)
Supplement: Table S3 — Gene set enrichment analysis of TNF-α treated human adipocyte microarray data according to gene ontology. (DOC) [file pone.0066515.s004.doc]

Table S3. Gene set enrichment analysis of TNF-α treated human adipocyte microarray data according to gene ontology.

| NAME | NES* | NOM p-value | FDR q-value |
| --- | --- | --- | --- |
| DEFENSE_RESPONSE | 3.164 | 0.011 | 0.244 |
| LOCOMOTORY_BEHAVIOR | 3.149 | 0.007 | 0.228 |
| CHEMOKINE_ACTIVITY | 3.135 | 0.005 | 0.213 |
| RESPONSE_TO_EXTERNAL_STIMULUS | 3.112 | 0.0167 | 0.204 |
| IMMUNE_SYSTEM_PROCESS | 3.103 | 0.0142 | 0.191 |
| CHEMOKINE_RECEPTOR_BINDING | 3.095 | 0.003 | 0.180 |
| RESPONSE_TO_OTHER_ORGANISM | 3.030 | < 0.0001 | 0.188 |
| RESPONSE_TO_VIRUS | 2.999 | 0.002 | 0.188 |
| CELL_CELL_SIGNALING | 2.987 | 0.018 | 0.181 |
| ION_HOMEOSTASIS | 2.930 | 0.010 | 0.188 |
| CELLULAR_HOMEOSTASIS | 2.915 | 0.008 | 0.184 |
| CELLULAR_CATION_HOMEOSTASIS | 2.911 | 0.010 | 0.176 |

**NES: normalized enrichment score*
