# Supplementary material for: Chemokine Expression in Inflamed Adipose Tissue Is Mainly Mediated by NF-κB
Source: PLoS One. 2013 Jun 18;8(6):e66515. doi: 10.1371/journal.pone.0066515 (PMC3688928; doi:10.1371/journal.pone.0066515)
Supplement: Table S4 — List of chemokine related genes significantly regulated by TNF-α treatment in 3T3-L1 adipocytes. (DOC) [file pone.0066515.s005.doc]

Table S4. List of chemokine related genes significantly regulated by TNF-α treatment in 3T3-L1 adipocytes.

| Gene symbol | Array Probe | Description | mRNA induction (fold change TNF vs. control) | p-value |
| --- | --- | --- | --- | --- |
| Ccl17 | A_51_P114462 | Mus musculus chemokine (C-C motif) ligand 17 (Ccl17), mRNA [NM_011332] | 3.1 | 1.181×10^-5^ |
| Ccl19 | A_51_P458258 | Mus musculus chemokine (C-C motif) ligand 19 (Ccl19), mRNA [NM_011888] | 18.7 | 4.296×10^-15^ |
|  | A_51_P458262 |  | 11.1 | 4.776×10^-15^ |
| Ccl2 | A_51_P286737 | Mus musculus chemokine (C-C motif) ligand 2 (Ccl2), mRNA [NM_011333] | 39.4 | 2.342×10^-15^ |
| Ccl20 | A_51_P408595 | Mus musculus chemokine (C-C motif) ligand 20 (Ccl20), mRNA [NM_016960] | 39.3 | 1.606×10^-10^ |
| Ccl24 | A_52_P18116 | Mus musculus chemokine (C-C motif) ligand 24 (Ccl24), mRNA [NM_019577] | -4.4 | 3.477×10^-7^ |
|  | A_51_P322640 |  | -2.5 | 7.325×10^-4^ |
| Ccl25 | A_51_P161265 | Mus musculus chemokine (C-C motif) ligand 25 (Ccl25), mRNA [NM_009138] | -1.1 | 1.341×10^-6^ |
|  | A_52_P326713 |  | 1.0 | 0.003 |
| Ccl27a | A_52_P618693 | Mus musculus chemokine (C-C motif) ligand 27A (Ccl27a), transcript variant 1, mRNA [NM_011336] | -1.9 | 5.803×10^-7^ |
| Ccl5 | A_52_P638459 | Mus musculus chemokine (C-C motif) ligand 5 (Ccl5), mRNA [NM_013653] | 122.0 | 5.854×10-^15^ |
|  | A_51_P485312 |  | 356.5 | 9.117×10^-17^ |
| Ccl7 | A_52_P208763 | Mus musculus chemokine (C-C motif) ligand 7 (Ccl7), mRNA [NM_013654] | 37.7 | 2.198×10^-12^ |
|  | A_51_P436652 |  | 35.1 | 8.160×10^-13^ |
| Ccl8 | A_51_P464703 | Mus musculus chemokine (C-C motif) ligand 8 (Ccl8), mRNA [NM_021443] | 100.2 | 6.055×10^-14^ |
| Ccl9 | A_51_P185660 | Mus musculus chemokine (C-C motif) ligand 9 (Ccl9), mRNA [NM_011338] | 37.3 | 6.277×10^-15^ |
| Ccrl2 | A_51_P232682 | Mus musculus chemokine (C-C motif) receptor-like 2 (Ccrl2), mRNA [NM_017466] | 6.7 | 1.961×10^-8^ |
| Cklf | A_52_P334593 | Mus musculus chemokine-like factor (Cklf), transcript variant 1, mRNA [NM_029295] | 4.9 | 2.730×10^-6^ |
|  | A_52_P652572 |  | 1.5 | 0.002 |
| Cx3cl1 | A_51_P196925 | Mus musculus chemokine (C-X3-C motif) ligand 1 (Cx3cl1), mRNA [NM_009142] | 3.8 | 8.836×10^-10^ |
| Cxcl1 | A_51_P363187 | Mus musculus chemokine (C-X-C motif) ligand 1 (Cxcl1), mRNA [NM_008176] | 32.4 | 3.306×10^-13^ |
| Cxcl10 | A_51_P432641 | Mus musculus chemokine (C-X-C motif) ligand 10 (Cxcl10), mRNA [NM_021274] | 147.2 | 1.239×10^-14^ |
| Cxcl11 | A_52_P676403 | Mus musculus chemokine (C-X-C motif) ligand 11 (Cxcl11), mRNA [NM_019494] | 237.0 | 7.205×10^-17^ |
| Cxcl12 | A_52_P685021 | Mus musculus chemokine (C-X-C motif) ligand 12 (Cxcl12), transcript variant 1, mRNA [NM_021704] | 2.8 | 2.239×10^-7^ |
|  | A_51_P172502 | Mus musculus chemokine (C-X-C motif) ligand 12 (Cxcl12), transcript variant 3, mRNA [NM_001012477] | 4.2 | 3.427×10^-9^ |
| Cxcl15 | A_52_P820196 | Mus musculus chemokine (C-X-C motif) ligand 15 (Cxcl15), mRNA [NM_011339] | -3.4 | 2.848×10^-4^ |
| Cxcl16 | A_51_P374203 | Mus musculus chemokine (C-X-C motif) ligand 16 (Cxcl16), mRNA [NM_023158] | 95.0 | 3.656×10^-14^ |
|  | A_52_P99888 |  | 28.3 | 4.6423×10^-15^ |
| Cxcl2 | A_51_P217463 | Mus musculus chemokine (C-X-C motif) ligand 2 (Cxcl2), mRNA [NM_009140] | 9.1 | 1.0129×10^-5^ |
| Cxcl5 | A_52_P295432 | Mus musculus chemokine (C-X-C motif) ligand 5 (Cxcl5), mRNA [NM_009141] | 31.3 | 1.619×10^-10^ |
| Cxcl9 | A_51_P461665 | Mus musculus chemokine (C-X-C motif) ligand 9 (Cxcl9), mRNA [NM_008599] | 29.0 | 1.684×10^-14^ |
| Ppbp | A_51_P428372 | Mus musculus pro-platelet basic protein (Ppbp), mRNA [NM_023785] | 2.7 | 0.0161 |
