# Supplementary material for: Chemokine Expression in Inflamed Adipose Tissue Is Mainly Mediated by NF-κB
Source: PLoS One. 2013 Jun 18;8(6):e66515. doi: 10.1371/journal.pone.0066515 (PMC3688928; doi:10.1371/journal.pone.0066515)
Supplement: Table S5 — Gene set enrichment analysis of TNF-α treated 3T3-L1 microarray data according to gene ontology. (DOC) [file pone.0066515.s006.doc]

Table S5. Gene set enrichment analysis of TNF-α treated 3T3-L1 microarray data according to gene ontology.

| NAME | NES* | NOM p-value | FDR q-value |
| --- | --- | --- | --- |
| CYTOKINE_ACTIVITY | 3.138 | < 0.0001 | < 0.0001 |
| RESPONSE_TO_EXTERNAL_STIMULUS | 3.121 | < 0.0001 | < 0.0001 |
| INFLAMMATORY_RESPONSE | 3.089 | < 0.0001 | < 0.0001 |
| G_PROTEIN_COUPLED_RECEPTOR_BINDING | 3.043 | < 0.0001 | 0.001 |
| LOCOMOTORY_BEHAVIOR | 3.032 | < 0.0001 | 9.043×10^-4^ |
| DEFENSE_RESPONSE | 3.029 | < 0.0001 | 7.536×10^-4^ |
| ION_HOMEOSTASIS | 3.025 | 0.002 | 8.103×10^-4^ |
| RESPONSE_TO_WOUNDING | 3.025 | < 0.0001 | 8.482×10^-4^ |
| RESPONSE_TO_CHEMICAL_STIMULUS | 2.970 | < 0.0001 | 0.002 |
| CHEMOKINE_ACTIVITY | 2.940 | < 0.0001 | 0.004 |

** NES: normalized enrichment score*
