# Supplementary material for: Chemokine Expression in Inflamed Adipose Tissue Is Mainly Mediated by NF-κB
Source: PLoS One. 2013 Jun 18;8(6):e66515. doi: 10.1371/journal.pone.0066515 (PMC3688928; doi:10.1371/journal.pone.0066515)
Supplement: Table S6 — Gene set enrichment analysis of TNF-α treated human adipocyte microarray data according to transcription factor response element present in the gene promoter. (DOC) [file pone.0066515.s007.doc]

Table S6. Gene set enrichment analysis of TNF-α treated human adipocyte microarray data according to transcription factor response element present in the gene promoter.

| NAME | NES* | NOM p-value | FDR q-value |
| --- | --- | --- | --- |
| V$NFKAPPAB_01 | 3.539 | < 0.0001 | 0.189 |
| V$IRF1_01 | 3.505 | < 0.0001 | 0.142 |
| V$CREL_01 | 3.376 | < 0.0001 | 0.314 |
| GGGNNTTTCC_V$NFKB_Q6_01 | 3.305 | < 0.0001 | 0.369 |
| V$IRF2_01 | 3.240 | < 0.0001 | 0.390 |
| V$NFKAPPAB65_01 | 3.193 | < 0.0001 | 0.410 |
| V$IRF1_Q6 | 3.183 | < 0.0001 | 0.362 |
| V$NFKB_Q6_01 | 3.174 | < 0.0001 | 0.326 |
| V$NFKB_C | 3.060 | < 0.0001 | 0.373 |
| V$STAT5A_01 | 3.014 | 0.0114 | 0.362 |

** NES: normalized enrichment score*
